# ¿Reforma de salud en Ecuador como modelo de éxito? Crítica al número especial de la Revista Panamericana de Salud Pública

**DOI:** 10.26633/RPSP.2017.148

**Published:** 2017-12-12

**Authors:** Irene Torres, Daniel López-Cevallos

**Affiliations:** 1 Aarhus University Copenhague, Dinamarca Fundación Octaedro, Quito, Ecuador Aarhus University, Copenhague, Dinamarca Fundación Octaedro, Quito, Ecuador; 2 Oregon State University Oregon University Oregon Oregon State United States Oregon State University Oregon, United States.

En mayo de este año, la *Revista Panamericana de Salud Pública* dedicó un número especial a promover el proceso más reciente de reforma de salud en Ecuador como un caso exitoso en la región. En el artículo editorial, la directora de la Organización Panamericana de la Salud (OPS), Carissa Etienne, plantea que los “sistemas de salud deben hallar modos innovadores para organizar y financiar los servicios de salud para poder progresivamente extender la cobertura, incrementar el acceso equitativo al cuidado, y proveer protección financiera, especialmente para los grupos poblacionales más vulnerables” (*traducción propia*).

En principio, llama la atención que se presente una visión sesgada del proceso de reforma, donde priman opiniones de funcionarios del gobierno y consultores del Ministerio de Salud Pública (MSP) y la OPS, en detrimento de contribuciones desde la academia y organizaciones de la sociedad civil. Adicionalmente, los autores plantean la atención primaria de salud (Modelo de Atención Integral de Salud, MAIS) como estrategia de reforma. Sin embargo, está claro que el modelo biomédico domina el sistema de salud en el Ecuador, siguiendo un patrón vertical y de cuidado episódico (1, 2), priorizando el tratamiento y la reparación, con enorme énfasis en la infraestructura y la especialización hospitalaria, por encima de la atención preventiva y de promoción de la salud (3, 4).

Aunque la cobertura mejoró en números, el análisis no arroja datos sobre la calidad del servicio ni su efectividad. En el discurso se plantea el MAIS como un cambio de enfoque “desde lo curativo hacia lo promocional y preventivo” a través de “la formación de técnicos de atención primaria en salud (TAPS)… y médicos familiares”, pero los autores no sustentan los “logros” mencionados como, por ejemplo, la idoneidad de los TAPS para fortalecer el MAIS, el impacto de la reorientación del año de servicio rural obligatorio hacia la promoción de la salud, o la capacidad del sistema de salud para dar una cobertura equitativa (5). Como ejemplo, las parteras tradicionales, que fueron importantes en la década anterior a 2006, fueron excluidas del sistema de salud sin considerar el aporte de sus conocimientos y experticia derivados de la práctica (6).

Parecería que una de las claves de la reforma ecuatoriana de salud es la recuperación de la rectoría del MSP, que ejerce como autoridad sanitaria. En ese sentido, no hay mayor sustento de que el MSP actúa como tal ni siquiera en programas tan elementales como el de alimentación escolar, que se basa en bebidas endulzadas y saborizadas artificialmente, barras energéticas y galletas altamente procesadas, contradiciendo las recomendaciones del mismo MSP de que los alimentos sean naturales y frescos (7). Más allá del etiquetado de alimentos, que de todas maneras adolece de errores intrínsecos a su diseño (8), ningún artículo presenta casos de la promoción que se supone que el MSP implementa. Adicionalmente, si la estrategia del etiquetado nutricional fuera suficiente y la inversión en salud, exitosa, ¿cómo se explica que la tasa de desnutrición crónica infantil continúe superando el 25%? (9).

De hecho, el artículo sobre el etiquetado evidencia que el discurso no se refleja en la realidad, pues los autores afirman que para fortalecer ese tipo de iniciativas es necesario “más presencia de organizaciones de la sociedad civil comprometidas con la defensa de los consumidores”, sin mencionar las restricciones legales y financieras que organizaciones de la sociedad civil han enfrentado en la última década, en especial después de la aplicación del Decreto Ejecutivo 16 (10). Este marco legal restrictivo de voces críticas tiene implicaciones en el sistema de salud, que los autores desafortunadamente no analizan.

Al presentar una visión sesgada del sistema de salud ecuatoriano, este número especial carece de perspectivas y datos que permitan conocer las implicaciones de los cambios realizados. Por tanto, es muy difícil que en el Ecuador y en otros países de la región se pueda entender la realidad y los retos de los sistemas de salud comparables. Habría sido pertinente que, como en otras ediciones de la revista, se incluyan referencias que permitan una mirada crítica de manera que se puedan reconocer e incluso corregir errores.

La reforma de salud implementada con un modelo curativo, vertical, basado en la cobertura universal de salud propuesta por la OPS, subordina el rol de la sociedad en la consecución del derecho a la salud. Sin partir de una visión de promoción y prevención profunda, real y participativa, que fomente e impulse la interculturalidad, plurinacionalidad y diversidad de saberes reconocidas en la Constitución del Ecuador, no se avanzará en la construcción de un sistema de salud que se adapte a la realidad ecuatoriana (1). Dejar de lado el análisis de estos factores continuará reforzando el uso de recetas preestablecidas en un marco de trabajo que ahonda, en lugar de subsanar, las condiciones de inequidad que caracterizan a los sistemas de salud ecuatoriano y de otros países latinoamericanos.
